# Isolation and characterization of multidrug-resistant *Proteus mirabilis* from diarrheic sika deer fawns in Northeast China

**DOI:** 10.3389/fcimb.2026.1866786

**Published:** 2026-10-02

**Authors:** Zhuqing Xue, Ziyu Liu, Xueliang Jiang, Qilin Wang, Runlai Cao, Yacong Li, Weiyang Wang, Peiwen Liu, Xiaorui Zhang, Cheng Chang, Hailong Huang, Xiaoxu Wang

**Affiliations:** 1 College of Veterinary Medicine, Jilin Agricultural University, Changchun, China; 2 Laboratory of Animal Infectious Disease Prevention and Control Technology, Institute of Special Animal and Plant Sciences, Chinese Academy of Agricultural Sciences, Changchun, China

**Keywords:** antimicrobial resistance, biofilm, fawn diarrhea, pathogenicity, *Proteus mirabilis*, sika deer, virulence genes

## Abstract

Diarrhea in sika deer fawns is a multifactorial disease that severely affects the development of deer farming in China. In the present study, we focused on *Proteus mirabilis* isolates recovered from diarrheic sika deer fawns in Heilongjiang, Jilin, and Liaoning provinces from 2022 to 2024. A total of 75 *P. mirabilis* isolates were identified using colony morphology and Gram staining together with 16S rRNA gene sequencing, type-strain-inclusive phylogenetic analysis, and atpD gene-targeted PCR as a supplementary molecular assay. Virulence genes and antimicrobial resistance genes were detected by PCR, antimicrobial susceptibility was determined using the broth microdilution method, and biofilm formation was evaluated by crystal violet staining. The pathogenic potential of representative isolates was further assessed using murine oral gavage and intraperitoneal challenge models, followed by observation of clinical signs and histopathological examination of the heart, liver, spleen, lung, and kidney. The isolates carried multiple virulence and antimicrobial resistance genes, exhibited multidrug-resistant phenotypes, and showed moderate to strong biofilm-forming capacity. Oral gavage with representative isolates induced obvious diarrhea in mice within two days, while intraperitoneal challenge caused clinical signs, mortality, and histopathological lesions in multiple organs. Whole-genome sequencing of the principal challenge strain Y24N1546 yielded a single circular chromosome of 4,073,229 bp. Genome-wide comparison with the *P. mirabilis* type strain ATCC 29906^T^ yielded an average nucleotide identity (ANI) of 98.94% and a digital DNA-DNA hybridization (dDDH) value of 93.4%; independent TYGS analysis also assigned Y24N1546 to *P. mirabilis*. Overall, these findings support *P. mirabilis* as an important bacterial pathogen associated with diarrheic sika deer fawns in Northeast China. Because fawn diarrhea is multifactorial, the etiological contribution of *P. mirabilis* should be interpreted together with other host, environmental, and management-related factors.

## 1 Introduction

Fawn diarrhea is a complex multifactorial disease in which bacterial pathogens can make an important contribution (Cho and Yoon, 2014). Pathogenic and multidrug-resistant *Escherichia coli* strains have previously been isolated from farm-raised diarrheic sika deer in Northeast China (Li et al., 2013). In our ongoing metagenomic analysis, fecal microbial composition also differed between healthy and diarrheic sika deer fawns, with increased abundance of potential bacterial pathogens, including *Escherichia coli* and *Proteus mirabilis*, in diarrheic animals. Together, these observations support further investigation of bacterial pathogens associated with fawn diarrhea.

*P. mirabilis*, a ubiquitous Gram-negative member of the Enterobacteriaceae family (Algammal et al., 2021), is an opportunistic pathogen with recognized clinical and veterinary significance. It has been associated with urinary, respiratory, wound, and digestive tract infections in humans and animals, including cattle, dogs, poultry, and fur-bearing animals (Armbruster et al., 2018; de Oliva et al., 2024; Humphries et al., 2021; Liu et al., 2023; Wang et al., 2010). In addition to its broad host range, *P. mirabilis* can harbor multiple virulence determinants, form biofilms, and acquire antimicrobial resistance genes (Jin et al., 2024), which may increase persistence in farming environments and reduce treatment efficacy.

Previous studies from different countries and host species have described antimicrobial resistance, virulence genes, biofilm formation, and genomic characteristics of *P. mirabilis* isolates from humans, dogs, poultry, cattle, and other animals (Armbruster et al., 2018; Sun et al., 2020; Sanches et al., 2020; Liu et al., 2023; de Oliva et al., 2024). However, deer-derived *P. mirabilis* remains poorly characterized, particularly in relation to fawn diarrhea. During bacteriological examination of diarrheic sika deer fawn fecal samples in our broader investigation, *P. mirabilis* was repeatedly recovered. This observation, together with previous reports of *P. mirabilis* from diarrheic animals in Northeast China (Sun et al., 2020; Hu et al., 2020), prompted us to systematically characterize these deer-derived isolates. Pathogenicity was subsequently evaluated using representative isolates in murine challenge models as an experimental assessment of their pathogenic potential.

This study aimed to isolate and characterize *P. mirabilis* from diarrheic sika deer fawns in Northeast China, focusing on molecular identification, virulence and antimicrobial resistance gene profiles, antimicrobial susceptibility, biofilm formation, and pathogenicity in murine oral gavage and intraperitoneal challenge models.

## 2 Materials and methods

### 2.1 Sample collection

From 2022 to 2024, 233 fecal samples were collected from diarrheic sika deer (*Cervus nippon*) fawns at 17 farms located in Heilongjiang, Jilin, and Liaoning provinces, major sika deer farming regions in Northeast China. Diarrheic fawns were generally less than one month old and were in the suckling to pre-weaning stage. Clinical signs included lethargy, reduced milk intake, and white or yellowish-green pasty or watery feces. Fecal samples were collected using sterile sampling tubes without transport medium, transported to the laboratory in an insulated container at approximately 4 °C, and processed within approximately 6 h after collection. The present study included 75 non-duplicate *P. mirabilis* isolates recovered from these diarrheic fecal samples and confirmed by molecular identification and phylogenetic analysis. When multiple colonies with similar morphology were recovered from the same sample, one representative colony was selected for purification and further characterization.

### 2.2 Bacterial isolation and morphological identification

Fecal samples were aseptically streaked onto Tryptic Soy Agar (TSA) plates (HopeBio™, China) using a three-zone streaking method. TSA was used for the initial recovery of a broad range of culturable bacteria from fecal samples, followed by purification and preliminary differentiation on Salmonella-Shigella (SS) selective agar (HopeBio™, China). The plates were incubated at 37 °C for 20–24 h. Single colonies suspected to be *Proteus* based on colony morphology, swarming characteristics, and Gram staining were purified and further confirmed by molecular methods.

### 2.3 16S rRNA gene PCR identification

Genomic DNA was extracted using a commercial bacterial genomic DNA extraction kit according to the manufacturer’s instructions. Universal bacterial 16S rRNA gene primers (27F: AGAGTTTGATCMTGGCTCAG; 1492R: TACGGYTACCTTGTTACGACTT; Sangon Biotech™, China) were used to amplify an approximately 1480 bp fragment. PCR amplification was performed on a ProFlex PCR System (Applied Biosystems™, USA) using 2× M5 HiPer Plus Taq HiFi PCR Mix (CW Bio™, China). The 25 µL reaction mixture contained 12.5 µL of master mix, 1 µL of each primer (10 µM), 2 µL of bacterial template, and 8.5 µL of ddH_2_O. The thermal cycling conditions were as follows: initial denaturation at 94 °C for 5 min; 35 cycles of 94 °C for 20 s, 60 °C for 30 s, and 72 °C for 90 s; and a final extension at 72 °C for 5 min. PCR products were analyzed by electrophoresis on a 1% agarose gel (JUNYI Dongfang™, China) and visualized under UV light (Bio-Rad™, USA). The standardized reference strain CMCCB49005 was used as a positive control for PCR amplification, and nuclease-free water was used as a negative control. The purified 16S rRNA PCR products were subjected to bidirectional Sanger sequencing, and the obtained sequences were compared with reference sequences in the NCBI database using BLAST.

### 2.4 atpD gene-targeted PCR

An atpD gene-targeted PCR assay was used as a supplementary molecular assay for the study isolates. The primer pair was F: GTATCATGAACGTTCTGGGTAC and R: TGAAGTGATACGCTCTTGCAG, with an expected amplicon of approximately 595 bp (Bi et al., 2013; Algammal et al., 2021). The PCR mixture and cycling conditions were identical to those used for 16S rRNA amplification, except that the extension step at 72 °C was adjusted to 45 s per cycle. Products were analyzed by electrophoresis on a 1% agarose gel.

### 2.5 Phylogenetic tree construction

To analyze genetic relationships among the isolates and improve species-level interpretation, a phylogenetic tree was constructed based on 16S rRNA gene sequences. Sequences of the study isolates were aligned with the P. mirabilis type strain ATCC 29906^T^, the laboratory reference strain P. mirabilis ATCC 12453, selected P. mirabilis reference sequences, and type strains of closely related Proteus species. Multiple-sequence alignment was performed using ClustalW in MEGA 12. Low-quality terminal regions and sites not shared across all sequences were removed, and all sequences were trimmed to the same 1,341-bp overlapping region before phylogenetic analysis. A Neighbor-Joining (NJ) tree was constructed, and branch support was evaluated using 1,000 bootstrap replicates. Bootstrap values ≥50% are shown at the nodes.

### 2.6 Whole-genome sequencing and genome-based taxonomic analysis

Whole-genome sequencing of the principal challenge strain Y24N1546 (sequencing sample ID Y1546) was performed by Comate Bioscience Co., Ltd. (Changchun, China) using Nanopore long-read and Illumina short-read sequencing data. Hybrid genome assembly was performed using Unicycler v0.4.7; the assembly workflow incorporated miniasm and Racon for long-read assembly, SPAdes for short-read assembly, and Pilon for genome polishing. For genome-based taxonomic confirmation, pairwise average nucleotide identity (ANI) between Y24N1546 and the P. mirabilis type strain ATCC 29906^T^ (GCA_000160755.1) was calculated using FastANI (Jain et al., 2018). Digital DNA-DNA hybridization (dDDH) was calculated using the Genome-to-Genome Distance Calculator (GGDC 3.0) with BLAST+, and the recommended Formula 2 value was used for species-level interpretation (Meier-Kolthoff et al., 2013). In addition, independent whole-genome taxonomic analysis was performed using the Type (Strain) Genome Server (TYGS) with default settings, including type-based species/subspecies clustering and whole-genome GBDP phylogenetic analysis (Meier-Kolthoff et al., 2022; Meier-Kolthoff and Göker, 2019). ANI values of approximately 95–96% and dDDH values of 70% were used as commonly accepted species delineation thresholds.

### 2.7 Detection of virulence genes

Primer sets for known *P. mirabilis* virulence genes (**Table 1**) were used as reported in the literature. Bacterial genomic DNA was used as the template. The 25 µL PCR mixture contained 12.5 µL of master mix, 1 µL of each primer (10 µM), 2 µL of template, and 8.5 µL of ddH_2_O. The cycling conditions were: 94 °C for 10 min; 35 cycles of 94 °C for 1 min, an annealing temperature of 56–59 °C for 1 min, and 72 °C for 30 s; followed by a final extension at 72 °C for 8 min. Amplification was confirmed by 1% agarose gel electrophoresis.

**Table 1:** Primer sequences for *Proteus mirabilis* virulence genes.

Genes	Oligonucleotide sequences	Amplified product (bp)	Annealing	References
*ureC*	F: GTTATTCGTGATGGGATGGG	375	56	(Elhoshi et al., 2023)
	R: ATAAAGGTGGTTACGCCA			
*mrpA*	F: ATTTCAGGAAACAAAAGATC	410	59	(Ramatla et al., 2024)
	R: TTCTTACTGATAAGAGATTG			
*zapA*	F: ACCGCAGGAAAACATATAGCCC	493	58	(Ramatla et al., 2024)
	R: GCGACTATCTTCCGCATAATCA			
*atfC*	F: AGAAAGGGATCCTACAAATTAA	472	57	(Hussein et al., 2020)
	R: TATAGCATGCATTTAAATTGCC			
*atfA*	F: CATAATTTCTAGACCTGCCCTAGCA	365	56	(Ramatla et al., 2024)
	R: CTGCTTGGATCCGTAATTTTTAACG			
*pmfA*	F: GGATCATCTATAATGAAACTG	534	58	(Ramatla et al., 2024)
	R: CTGATAATCAACTTGGAAGTT			
*ucaA*	F: GTAAAGTTGTTGCGCAAAC	587	55	(Ramatla et al., 2024)
	R: TTGAGCCACTGTGGATACA			
*rsbA*	F: TCGATTTCAGTGTTTGGCCAT	647	55	(Pathirana et al., 2018)
	R: CCGAGCTTCATCATGGCTG			

### 2.8 Detection of antimicrobial resistance genes

Similarly, primer sets for various AMR genes (**Table 2**) were used as reported. The PCR mixture was identical to that used for virulence-gene detection. The thermal profile was: 94 °C for 10 min; 35 cycles of 94 °C for 1 min, annealing at 51–62 °C for 1 min, and 72 °C for 30 s; with a final extension at 72 °C for 8 min. Products were visualized on 1% agarose gels.

**Table 2:** Primer sequences for *Proteus mirabilis* antimicrobial resistance genes.

Type	Genes	Oligonucleotide sequences	Amplified product (bp)	Annealing	References
Tetracyclines	*tetA*	F:GCTACATCCTGCTTGCCTTC	210	57	(Guo et al., 2019)
		R:CATAGATCGCCGTGAAGAGG			
	*tetB*	F:ACAGAAAGCTTATTATATAAC	186	51	(Liu et al., 2023)
		R:TGGCGTGTCTATGATGTTCAC			
	*tetO*	F:GATGGCATACAGGCACAGACC	418	57	(Galani et al., 2012)
		R:GCCCAACCTTTTGCTTCACTA			
Aminoglycoside	*rmtA*	F:CTAGCGTCCAT CCTTTCCTC	635	57	(Galani et al., 2012)
		R:TTTGCTTCCATGCCCTTGCC			
	*ant (3 ″)-I*	F:TGATTTGCTGGTTACGGTGAC	284	53	(Galani et al., 2012)
		R:CGCTATGTTCTCTTGCTTTTG			
	*strA*	F:TGCTCCTCTTCTCCATCC	197	54	(Harada et al., 2014)
		R:CGCCGCCAATATGTTCTA			
Sulfonamides	*sul2*	F:CGGCATCGTCAACATAACCT	721	56	(Guo et al., 2019)
		R:TGTGCGGATGAAGTCAGCTC			
	*sul3*	F:AGATGTGATTGTTTGGGAGC	443	56	(Guo et al., 2019)
		R:TAGTTGTTTCTGGATTAGAGCCT			
	*pmi*	F:TGCCTTGAGACAGGGTAAGC	739	56	(Elhoshi et al., 2023)
		R:GCCTTAACAAGTCCCATTCG			
β-Lactamase Inhibitors	*blaTEM*	F:ATCAGCAATAAACCAGC	516	54	(Algammal et al., 2021)
		R:CCCCGAAGAACGTTTTC			
	*blaCTX-M*	F:ATGTGCAGYACCAGTAARGTKATGGC	593	54	(Algammal et al., 2021)
		R:TGGGTRAARTARGTSACCAGAAYCAGCGG			
Carbapenem	*blaOXA-1*	F:ATATCTCTACTGTTGCATCTCC	619	54	(Guo et al., 2019)
		R:AAACCCTTCAAACCATCC			
	*blaKPC*	F:ATGTCACTGTATCGCCGTCT	692	55	(Algammal et al., 2021)
		R:TTACTGCCCGTTGACGCCC			
Polymyxins	*mcr-1*	F:CGGTCAGTCCGTTTGTTC	320	52	(Sun et al., 2020)
		R:CTTGGTCGGTCTGTAGGG			
	*mcr-9*	F:GGTATCCTTCCTGCCATC	929	60	(Sun et al., 2020)
		R:TGATTCACCATGATCGGAC			
Fluoroquinolones	*qnrS*	F:GCAGCTTCATTGAACAGGGT	428	54	(Lv et al., 2022)
		R:TCTAAACCGTCGAGTTCGGGC			
	*oqxA*	F:GATCAGTCAGTGGGATAGTTT	670	51	(Lv et al., 2022)
		R:TACTGGGGGTTTAGTGTATTA			

### 2.9 Antimicrobial susceptibility testing

The minimum inhibitory concentrations (MICs) of 18 antibiotics across 9 classes (selected per CLSI M100-ED33 (Humphries et al., 2021): Ceftriaxone-CRO, Ampicillin-AMP, Piperacillin-PIP, Imipenem-IPM, Meropenem-MEM, Polymyxin B-PB, Gentamicin-GEN, Tobramycin-TOB, Kanamycin-KAN, Tetracycline-TET, Doxycycline-DOX, Norfloxacin-NOR, Gatifloxacin-GAT, Sulfisoxazole-SIZ, Trimethoprim-TMP, Chloramphenicol-CHL, Florfenicol-FFC, Fosfomycin Sodium-FOS; Solarbio, China) were determined using the broth microdilution method. Results were interpreted according to CLSI guidelines (M100-ED33) (Humphries et al., 2021).

### 2.10 Biofilm formation assay

The biofilm-forming capacity of the isolates was quantified using the crystal violet staining method as previously described (Liu et al., 2023). The cut-off optical density value was defined as ODc = mean OD of the negative control + 3 standard deviations. Biofilm formation was classified as follows: OD ≤ ODc, non-biofilm producer; ODc < OD ≤ 2ODc, weak biofilm producer; 2ODc < OD ≤ 4ODc, moderate biofilm producer; OD > 4ODc, strong biofilm producer.

### 2.11 Murine pathogenicity assay

Specific pathogen-free (SPF) BALB/c mice, 7 weeks old and weighing 18 ± 2 g, were used in this study, with equal numbers of males and females in each group. Mice were randomly assigned to different experimental groups. The group size was determined based on previous bacterial infection models and preliminary experiments, while also following the principle of reducing animal use under ethical requirements.

To evaluate intestinal pathogenic potential, an oral gavage model was performed using representative isolates. Bacterial suspensions were prepared from 24-h TSB cultures and adjusted to approximately 1 × 10^9^ CFU per 200 μL. Eight mice were included in each group, and the control group received sterile saline. Mice were monitored for diarrhea, lethargy, reduced activity, anorexia, and ruffled fur according to predefined clinical observation criteria.

To further compare systemic virulence among representative isolates, an intraperitoneal challenge model was used. Representative isolates with different virulence gene profiles were selected for this assay. Bacterial suspensions were prepared from 24-h TSB cultures and adjusted to the indicated doses based on preliminary experiments. Groups of six mice were challenged by intraperitoneal injection with 200 μL containing either 1 × 10^9^ CFU or 2 × 10^8^ CFU. Mice were monitored for mortality and clinical signs over a 10-day period. Humane endpoints included severe lethargy, persistent recumbency, inability to access food or water, or moribund status. Dead or euthanized mice from the 1546 challenge group were subjected to necropsy, and the heart, liver, spleen, lung, and kidney were collected for histopathological examination.

## 3 Results

### 3.1 Isolation and molecular identification of *P. mirabilis*

A total of 75 non-duplicate isolates were identified as *P. mirabilis* from diarrheic sika deer fawn fecal samples and retained for subsequent analyses. The isolates showed typical Gram-negative short-rod morphology and exhibited swarming motility on TSA, while swarming was inhibited on SS agar. These phenotypic characteristics were used for preliminary screening and were followed by molecular identification.

PCR amplification of the 16S rRNA gene produced an approximately 1,480-bp fragment in all 75 isolates. Bidirectional Sanger sequencing followed by BLAST analysis showed high similarity to *P. mirabilis* reference sequences. The atpD gene-targeted PCR yielded the expected single amplicon of approximately 595 bp in the isolates. Species-level identification was further evaluated using a type-strain-inclusive 16S rRNA phylogenetic analysis. Representative colony morphology, Gram staining, and 16S rRNA and atpD PCR electrophoresis results are provided in **Supplementary Figures S1–S3**.

### 3.2 Phylogenetic analysis

The 16S rRNA phylogenetic tree (**Figure 1**) showed that all 75 study isolates were positioned within the *P. mirabilis* lineage containing the type strain ATCC 29906^T^, the laboratory reference strain ATCC 12453, and additional *P. mirabilis* reference sequences, and were separated from the type strains of closely related *Proteus species* included for comparison.

**Figure 1:**
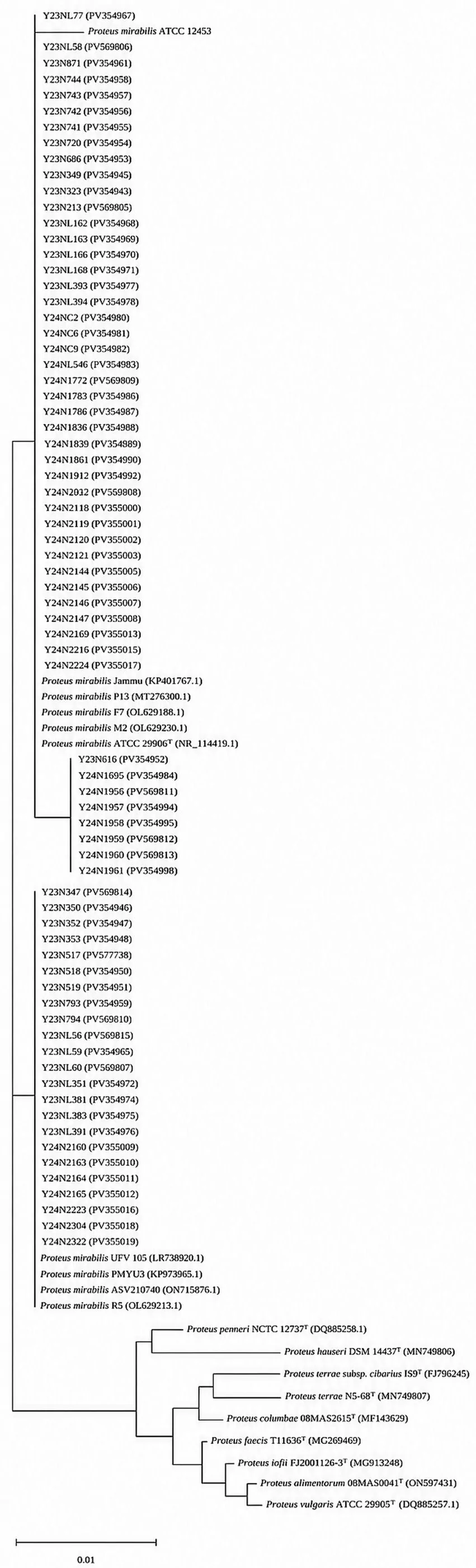
Neighbor-Joining phylogenetic tree based on 1,341-bp 16S rRNA gene sequences of 75 *Proteus mirabilis* isolates and reference strains. The tree was constructed in MEGA 12 using the Neighbor-Joining method with 1,000 bootstrap replicates. Bootstrap values ≥50% are shown at the nodes. GenBank accession numbers, where available, are shown in parentheses. Superscript T indicates a type strain. P. mirabilis ATCC 29906^T^ was included as the type strain, whereas P. mirabilis ATCC 12453 was included as the laboratory reference strain. Type strains of closely related *Proteus* species were included for species-level comparison. The scale bar represents 0.002 substitutions per site.

### 3.3 Whole-genome confirmation of the principal challenge strain Y24N1546

Whole-genome sequencing of Y24N1546 generated 1.75 Gb of clean Oxford Nanopore data comprising 206,770 reads, with a read N50 of 15,528 bp. Hybrid polishing of the long-read assembly with Illumina data yielded a single circular chromosome of 4,073,229 bp with a G+C content of 39.11%. FastANI comparison with the *P. mirabilis* type strain ATCC 29906^T^ (GCA_000160755.1) yielded an ANI value of 98.94%; 1,216 of 1,357 query fragments were mapped (89.61%). GGDC analysis produced a recommended Formula 2 dDDH value of 93.4% (95% model-based confidence interval, 91.5–94.8%) and a G+C difference of 0.50%. TYGS independently assigned Y24N1546 to *P. mirabilis*; Y24N1546 and the *P. mirabilis* type strain ATCC 29906^T^ were placed in the same species and subspecies clusters, and the whole-genome GBDP phylogeny had an average branch support of 97.4% (**Supplementary Figure S4**). Together, these genome-based analyses strongly support the identification of Y24N1546 as *P. mirabilis*.

### 3.4 Virulence gene profiles

The distribution and combination of detected virulence genes among the 75 *P. mirabilis* isolates are summarized in **Table 3**. The frequency of individual virulence genes is shown in **Figure 2**.

**Table 3:** Virulence gene combinations among the 75 *P. mirabilis* isolates.

Virulence gene combination	Number of strains	Percentage(%)
*atfA, atfC, ureC, zapA, rsbA, ucaA*	12	16.00%
*atfA, pmfA, ucaA, atfC, ureC, zapA*	2	2.67%
*atfA, atfC, ureC, zapA, rsbA*	23	30.67%
*atfA, pmfA, atfC, ureC, zapA*	9	12.00%
*atfA, atfC, ureC, zapA, ucaA*	2	2.67%
*atfA, pmfA, ureC, zapA*	8	10.67%
*pmfA, atfC, ureC, zapA*	4	5.33%
*atfA, rsbA, ureC, zapA*	2	2.67%
*atfA, atfC, ureC, zapA*	1	1.33%
*atfA, ureC, zapA*	2	2.67%
*ureC, zapA*	4	5.33%
No target virulence genes detected	6	8.00%

**Figure 2:**
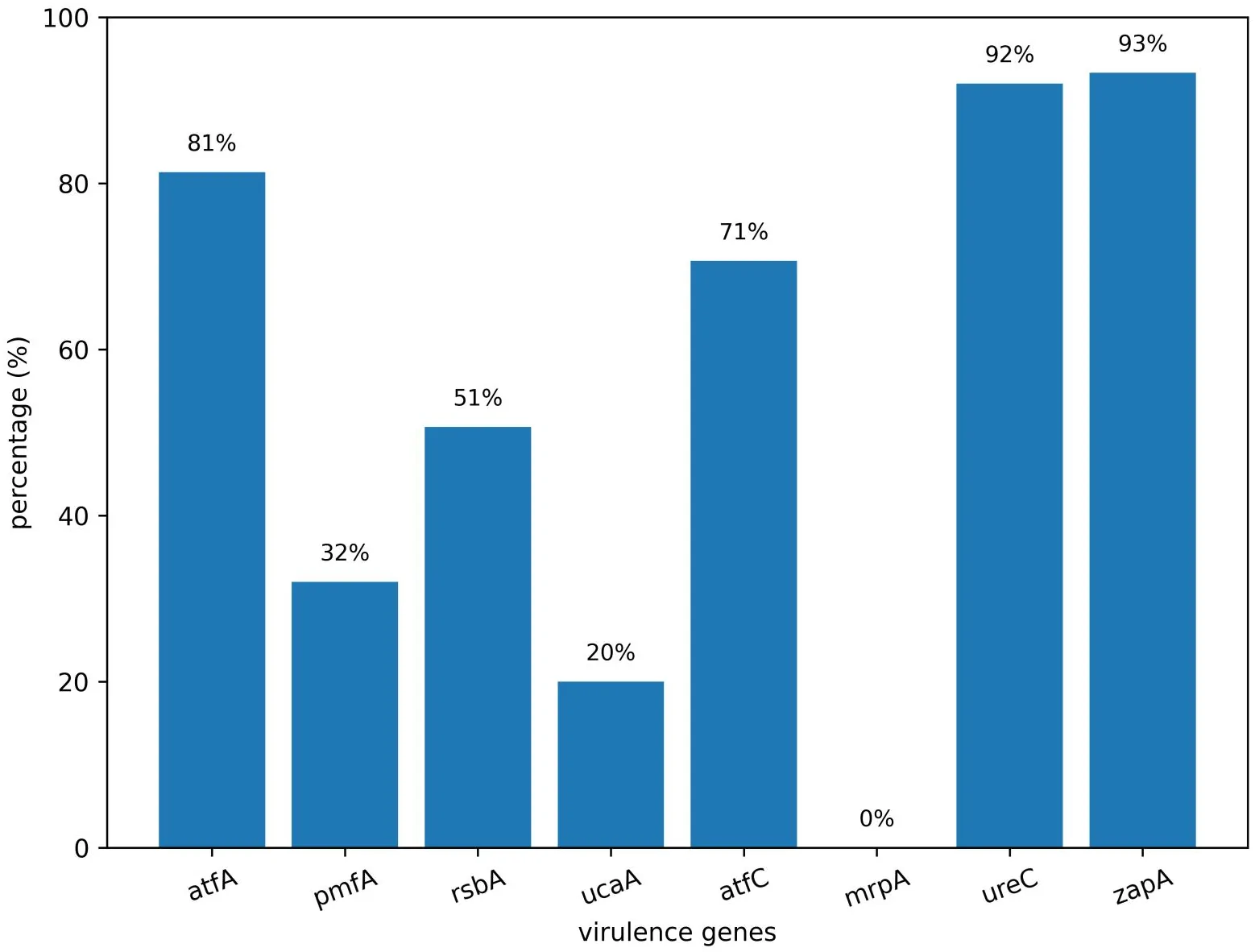
Frequency of individual virulence genes among the 75 *P. mirabilis* isolates.

### 3.5 Antimicrobial resistance gene profiles

The distribution and selected combinations of detected AMR genes among the 75 *P. mirabilis* isolates are summarized in **Table 4**. The frequency of individual AMR genes is shown in **Figure 3**.

**Table 4:** Selected combinations of antimicrobial resistance genes among the 75 *P. mirabilis* isolates.

Drug resistance gene combination	Strains ID	Percentage (%)
*tetA tetB tetO rmtA ant(3”)-I strA sul2 blaTEM blaCTX-M blaOXA-1 mcr-9*	Y24N1695	1.33%
*tetA tetB tetO rmtA ant(3”)-I strA sul2 sul3 blaTEM blaCTX-M blaOXA-1*	Y24N2304, Y24N2322	2.67%
*tetA tetB ant(3”)-I strA sul2 sul3 blaTEM blaCTX-M blaOXA-1 blaKPC*	Y23N213	1.33%
*tetA tetO rmtA ant(3”)-I strA sul2 sul3 blaTEM blaCTX-M blaOXA-1*	Y24N1772	1.33%
*tetA tetO rmtA ant(3”)-I strA sul2 blaTEM blaCTX-M blaOXA-1 mcr-9*	Y24N1786	1.33%
*tetO rmtA ant(3”)-I strA sul2 sul3 blaTEM blaCTX-M blaOXA-1 mcr-9*	Y24N1836, Y24N1839	2.67%
*tetA tetO rmtA ant(3”)-I strA sul2 blaTEM blaCTX-M blaOXA-1*	Y24NC2, Y24N1861	2.67%
*tetO rmtA ant(3”)-I strA sul2 sul3 blaTEM blaCTX-M blaOXA-1*	Y24N1957	1.33%
*tetA tetO rmtA ant(3”)-I strA sul2 blaTEM blaCTX-M blaOXA-1*	Y24N2146	1.33%
*tetA tetB ant(3”)-I strA sul2 sul3 blaOXA-1 blaKPC*	Y23N323	1.33%

**Figure 3:**
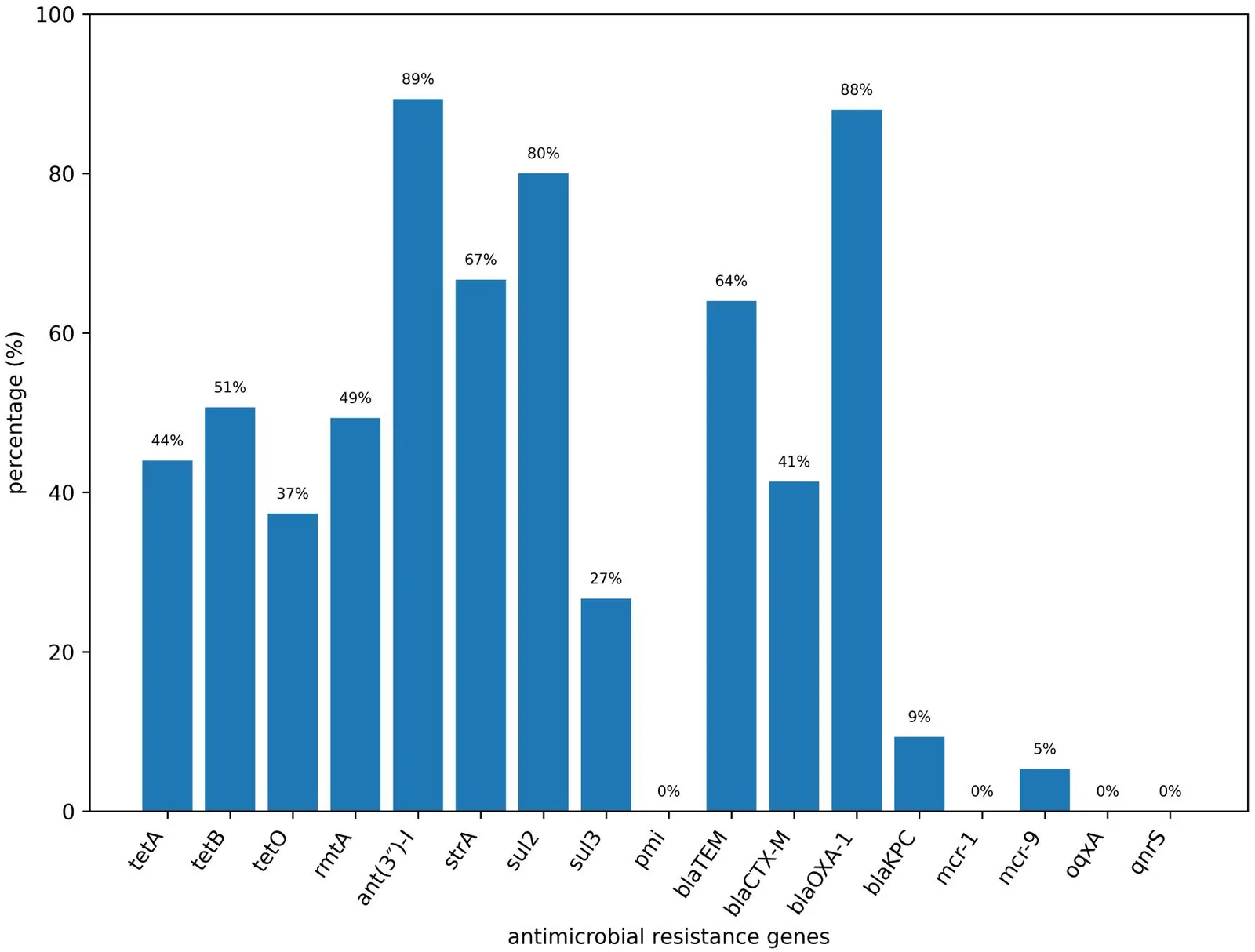
Frequency of individual antimicrobial resistance genes among the 75 *P. mirabilis* isolates.

### 3.6 Antimicrobial susceptibility

The MIC results indicated widespread multidrug resistance among the 75 *P. mirabilis* isolates. Susceptibility was partially retained to imipenem, whereas susceptibility to meropenem and fluoroquinolones remained markedly reduced (**Table 5**).

**Table 5:** Antibiogram of the 75 *P. mirabilis* isolates.

Antimicrobial classes	Antimicrobial agents	R, n (%)	I, n (%)	S, n (%)
β-Lactams	Ceftriaxone	75 (100.00%)	0	0
	Ampicillin	75 (100.00%)	0	0
	Piperacillin	75 (100.00%)	0	0
Carbapenem	Imipenem	14 (18.67%)	10 (13.33%)	51 (68.00%)
	Meropenem	61 (81.33%)	10 (13.33%)	4 (5.33%)
Polymyxins	Polymyxin B	75 (100.00%)	0	0
Aminoglycosides	Gentamicin	75 (100.00%)	0	0
	Tobramycin	75 (100.00%)	0	0
	Kanamycin	75 (100.00%)	0	0
Tetracyclines	Tetracycline	75 (100.00%)	0	0
	Doxycycline	70 (93.33%)	5 (6.67%)	0
Quinolones	Norfloxacin	50 (66.67%)	19 (25.33%)	6 (8.00%)
	Gatifloxacin	59 (78.67%)	13 (17.33%)	3 (4.00%)
Folate pathway inhibitors	Sulfisoxazole	75 (100.00%)	0	0
	Trimethoprim	75 (100.00%)	0	0
Amphenicols	Chloramphenicol	75 (100.00%)	0	0
	Florfenicol	75 (100.00%)	0	0
Fosfomycins	Fosfomycin Sodium	55 (73.33%)	20 (26.67%)	0

### 3.7 Biofilm formation capacity

All 75 *P. mirabilis* isolates demonstrated the ability to form biofilms. The cut-off optical density value (ODc) was defined as the mean OD of the negative control plus three standard deviations and was calculated as 0.085. Based on this ODc, 3 isolates (4.00%) were classified as moderate biofilm formers (0.17 < OD590 ≤ 0.34), whereas 72 isolates (96.00%) were classified as strong biofilm formers (OD590 > 0.34).

### 3.8 Murine pathogenicity

In the oral gavage model, mice administered strain Y24N1546 or the mixed isolate suspension developed obvious diarrhea within two days, while the blank control group showed no comparable clinical signs. In the intraperitoneal challenge model, mice challenged with selected isolates displaying different virulence gene profiles developed clinical signs including lethargy, anorexia, ruffled fur, reduced activity, and diarrhea. The number of deaths and time to death for each challenge group are detailed in **Table 6**. Histopathological examination of mice challenged with Y24N1546 revealed lesions in the heart, liver, spleen, lung, and kidney, supporting systemic pathogenic effects after intraperitoneal infection (**Figure 4**).

**Table 6:** Pathogenicity of selected *P. mirabilis* isolates in mice.

Number of virulence genes (combination type)	Strain ID	Challenge dose (CFU)	Number of dead mice	Time to death (h)
6 (*atfA,atfC,ureC,zapA,ucaA,rsbA*)	Y24N1546	1×10^9^	6/6	16
		2×10^8^	6/6	16
6 (*atfA,atfC,ureC,zapA,ucaA, pmfA*)	Y23NL383	1×10^9^	6/6	20
		2×10^8^	0/6	-
5 (*atfA,atfC,ureC,zapA,rsbA*)	Y24N1695	1×10^9^	6/6	18
		2×10^8^	5/6	18
5 (*atfA,atfC,ureC,zapA,pmfA*)	Y23NL381	1×10^9^	6/6	22
		2×10^8^	0/6	-
5 (*atfA,atfC,ureC,zapA,ucaA*)	Y24N2160	1×10^9^	6/6	19
		2×10^8^	5/6	21
4 (*atfA,atfC,ureC,zapA*)	Y24N2224	1×10^9^	4/6	20
		2×10^8^	2/6	23

**Figure 4:**
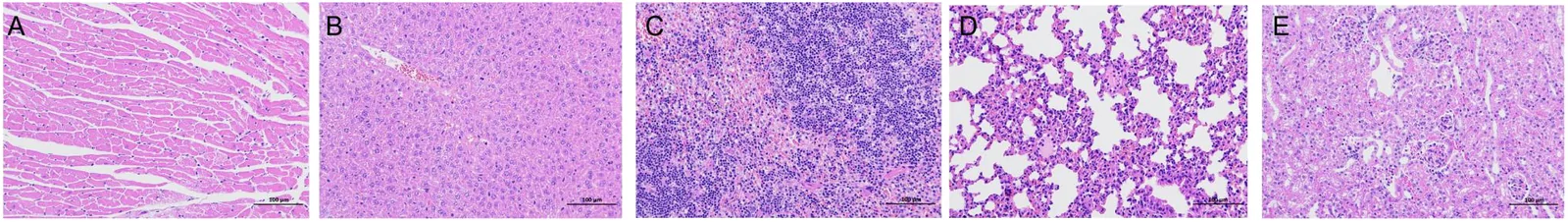
Histopathological sections of heart, liver, spleen, lung, and kidney tissues after challenge with strain Y24N1546. H&E staining; scale bar = 100 μm. **(A)** heart; **(B)** liver; **(C)** spleen; **(D)** lung; **(E)** kidney.

## 4 Discussion

*P. mirabilis* is a clinically important opportunistic pathogen that can cause urinary tract, digestive tract, respiratory, wound, and systemic infections in humans and animals. It is a well-recognized cause of catheter-associated urinary tract infection and has also been implicated in foodborne disease (Armbruster et al., 2018; Wang et al., 2010). In veterinary medicine, *P. mirabilis* has been reported from multiple animal hosts, including poultry, dogs, cattle, and fur-bearing animals (de Oliva et al., 2024; Jin et al., 2024; Liu et al., 2023). During bacteriological examination of fecal samples from diarrheic sika deer fawns in Northeast China, *P. mirabilis* was repeatedly recovered, prompting the systematic characterization of these isolates in the present study. The isolates were examined for molecular characteristics, virulence and antimicrobial resistance gene profiles, antimicrobial susceptibility, and biofilm formation. Representative isolates were subsequently selected according to their molecular and virulence-gene characteristics for murine challenge experiments, which further demonstrated their pathogenic potential. Type-strain-inclusive 16S rRNA phylogenetic analysis supported the assignment of all 75 isolates to *P. mirabilis*. Whole-genome analysis provided higher-resolution confirmation for the principal challenge strain Y24N1546: ANI (98.94%) and dDDH (93.4%) relative to the *P. mirabilis* type strain ATCC 29906^T^ exceeded accepted species boundaries, and TYGS independently assigned Y24N1546 to the same *P. mirabilis* species and subspecies clusters as the type strain. Together, these results strengthen the taxonomic basis for interpretation of the challenge experiments.

Virulence genes encode determinants involved in adhesion, motility, urease production, immune evasion, and colonization. In this study, the carriage of virulence genes varied among isolates, and representative strains with higher virulence gene burden tended to show greater mortality and shorter survival time in the murine intraperitoneal challenge model. Oral gavage with representative isolates also induced diarrhea in mice, supporting intestinal pathogenic potential. However, because gene knockout and complementation assays were not performed, the detected gene combinations should be interpreted as being associated with increased pathogenicity rather than as directly conferring enhanced virulence on their own. These data provide a useful basis for selecting target strains for future mechanistic studies.

The antimicrobial resistance results revealed a concerning multidrug-resistant phenotype among deer-derived study isolates. Several resistance genes were detected by PCR, including genes related to β-lactams, aminoglycosides, tetracyclines, sulfonamides, and polymyxins. However, phenotypic resistance was not always fully explained by the detected genes. This discrepancy may be associated with resistance determinants not included in the PCR panel, chromosomal mutations, altered membrane permeability, efflux pump activity, biofilm formation, or differences in gene expression. Therefore, the PCR-based resistance gene profile should be considered a partial characterization of the resistome, and future whole-genome sequencing and expression analyses are needed to clarify the underlying mechanisms.

Antimicrobial susceptibility testing (AST) revealed a severe resistance phenotype among the *P. mirabilis* isolates. The susceptibility rate to imipenem was 68.00%, whereas susceptibility to meropenem was only 5.33%. Susceptibility to norfloxacin and gatifloxacin was 8.00% and 4.00%, respectively. Doxycycline and fosfomycin showed intermediate susceptibility in a proportion of isolates, while no susceptible isolates were observed for the remaining tested agents. Compared with reports from other animal- and human-derived *P. mirabilis* collections, the resistance profile observed in the present study was generally more severe. For example, Liu et al. reported imipenem and meropenem resistance rates of 70.67% and 29.33%, respectively, among isolates from stray dogs (Liu et al., 2023). Chen et al. reported imipenem and meropenem susceptibility rates of 93.4% and 100%, respectively, among human urinary isolates (Chen et al., 2018), while Sanches et al. reported 100% susceptibility to imipenem among isolates from a poultry slaughterhouse (Sanches et al., 2020). Carbapenems, including imipenem, ertapenem, meropenem, and doripenem, are regarded in many settings as critically important agents for severe infections caused by ESBL-producing Enterobacteriaceae (Girlich et al., 2020), making the reduced susceptibility observed in the present collection particularly concerning.

For fluoroquinolones, Yu et al. reported norfloxacin susceptibility rates of 52% in poultry-derived isolates and 65% in human-derived isolates, both substantially higher than the 8.00% observed in the present collection (Yu et al., 2021). Differences were also evident for other antimicrobial classes. Yu et al. reported susceptibility rates of 100%, 38%, and 56% to ceftriaxone, ampicillin, and piperacillin, respectively, among poultry isolates (Yu et al., 2021). Similarly, Sanches et al. reported resistance rates exceeding 70% for ampicillin and 45% for ceftriaxone (Sanches et al., 2020), while Liu et al. reported 36% resistance to ampicillin (Liu et al., 2023). Aminoglycoside susceptibility in the present collection was also markedly reduced relative to several previous reports. Yu et al. found gentamicin and tobramycin susceptibility rates of 56% and 88%, respectively (Yu et al., 2021), and Liu et al. reported only 12% gentamicin resistance (Liu et al., 2023). The resistance profile observed here was more comparable to that reported by Lv et al. in isolates from fur-bearing animals (Lv et al., 2022). In addition, phenotypic resistance to polymyxins in the absence of detected mcr genes may reflect chromosomal mechanisms, including lipopolysaccharide modification mediated by the arnBCADTEF operon and eptB (Aghapour et al., 2019). Collectively, these AST findings underscore the need for prudent antimicrobial use and continued resistance surveillance in sika deer production systems.

Biofilm formation constitutes a key defensive strategy, enhancing environmental persistence and intrinsic antibiotic tolerance while facilitating horizontal gene transfer (Yin et al., 2019; Nickel et al., 1985; de la Fuente-Núñez et al., 2013). All 75 *P. mirabilis* isolates formed biofilms; 3 isolates (4.00%) were moderate biofilm formers and 72 isolates (96.00%) were strong biofilm formers. This is consistent with previous observations that multidrug-resistant isolates often form more robust biofilms than susceptible isolates (Liu et al., 2023; Filipiak et al., 2020) and that biofilms can facilitate the persistence and dissemination of antimicrobial resistance determinants (Savage et al., 2013). The strong biofilm-forming phenotype observed in this collection may act together with the broad AMR profile to increase persistence and complicate treatment. Strategies targeting biofilm disruption may therefore provide a useful adjunct for controlling infection and limiting the spread of resistance determinants.

This study has several limitations. First, only diarrheic fawn samples were included, and matched healthy controls were not systematically analyzed. Second, whole-genome sequencing was performed for the principal challenge strain Y24N1546 rather than for all *P. mirabilis* isolates; consequently, genome-level epidemiological and strain-to-strain comparisons could not be performed across the full collection. Third, antimicrobial resistance genes were examined using targeted PCR assays rather than comprehensive genome-wide resistome and expression analyses. Finally, the murine intraperitoneal challenge model was used to compare systemic virulence under experimental conditions and does not fully reproduce natural intestinal infection in sika deer fawns.

## 5 Conclusion

In conclusion, this study characterized 75 multidrug-resistant *P. mirabilis* isolates recovered from diarrheic sika deer fawns in Northeast China. Genome-based ANI, dDDH, and TYGS analyses provided strong species-level confirmation of the principal challenge strain Y24N1546. The isolates carried multiple virulence and antimicrobial resistance genes, exhibited multidrug-resistant phenotypes, and showed moderate to strong biofilm-forming capacity. Representative isolates induced diarrhea following oral gavage and caused systemic disease, mortality, and histopathological lesions following intraperitoneal challenge in mice. These findings support *P. mirabilis* as an important bacterial pathogen associated with diarrheic sika deer fawns in Northeast China, while its contribution should be interpreted in the context of the multifactorial nature of fawn diarrhea.

## Data Availability

The 16S rRNA gene sequences corresponding to the P. mirabilis isolates analyzed in this study have been deposited in GenBank; individual accession numbers are provided in **Figure 1**. The whole-genome sequence of P. mirabilis strain Y24N1546 has been deposited at DDBJ/ENA/GenBank under the accession number JCDFTO000000000. The version described in this paper is JCDFTO010000000. The associated BioProject and BioSample accession numbers are PRJNA1519118 and SAMN62713668, respectively. The atpD PCR products were not sequenced. Other relevant data are included in this article and its **Supplementary Material**.

## Supplementary material

Supplementary Figure S1 Representative colony morphology and Gram staining of *Proteus mirabilis*. **(A)** Colony morphology on Tryptic Soy Agar (TSA). **(B)** Colony morphology on Salmonella–Shigella (SS) agar. **(C)** Gram-stained cells of *P. mirabilis*.

Supplementary Figure S2 Agarose gel electrophoresis of 16S rRNA gene PCR products of *Proteus mirabilis*. Lanes 1–11, representative isolates; lane 12, negative control.

Supplementary Figure S3 Agarose gel electrophoresis of atpD gene PCR products of *Proteus mirabilis*. Lanes 1–11, representative isolates; lane 12, negative control.

Supplementary Figure S4 Whole-genome GBDP phylogenetic tree of *Proteus mirabilis* strain Y24N1546 and closely related *Proteus* strains. The phylogenetic tree was generated from whole-genome GBDP distances using TYGS. Branch support values are shown at the nodes. Strain Y24N1546 clustered with the *P. mirabilis* type strain ATCC 29906^T^.

## References

[B1] Aghapour Z. Gholizadeh P. Ganbarov K. Bialvaei A. Z. Mahmood S. S. Tanomand A. . (2019). Molecular mechanisms related to colistin resistance in Enterobacteriaceae. Infect. Drug Resist. 12, 965–975. doi: 10.2147/IDR.S199844 31190901 PMC6519339

[B2] Algammal A. M. Hashem H. R. Alfifi K. J. Hetta H. F. Sheraba N. S. Ramadan H. . (2021). atpD gene sequencing, multidrug resistance traits, virulence-determinants, and antimicrobial resistance genes of emerging XDR and MDR-Proteus mirabilis. Sci. Rep. 11, 9476. doi: 10.1038/s41598-021-88861-w 33947875 PMC8096940

[B3] Armbruster C. E. Mobley H. L. T. Pearson M. M. (2018). Pathogenesis of Proteus mirabilis infection. EcoSal Plus 8 (1), 292. doi: 10.1128/ecosalplus.ESP-0009-2017 29424333 PMC5880328

[B4] Bi S. L. Tang S. Z. Wu X. Y. Chen S. Y. (2013). Quantitative detection of Proteus species by real-time polymerase chain reaction using SYBR Green I. Ann. Microbiol. 63, 1205–1208. doi: 10.1007/s13213-012-0519-330311153

[B5] Chen D. Zhang Y. Huang J. Liang X. Zeng T. Lan C. . (2018). The analysis of microbial spectrum and antibiotic resistance of uropathogens isolated from patients with urinary stones. Int. J. Clin. Pract. 72, e13205. doi: 10.1111/ijcp.13205 29790623

[B6] Cho Y. I. Yoon K. J. (2014). An overview of calf diarrhea - infectious etiology, diagnosis, and intervention. J. Vet. Sci. 15, 1–17. doi: 10.4142/jvs.2014.15.1.1 24378583 PMC3973752

[B7] de la Fuente-Núñez C. Reffuveille F. Fernández L. Hancock R. E. (2013). Bacterial biofilm development as a multicellular adaptation: antibiotic resistance and new therapeutic strategies. Curr. Opin. Microbiol. 16, 580–589. doi: 10.1016/j.mib.2013.06.013 23880136

[B8] de Oliva B. H. D. do Nascimento A. B. de Oliveira J. P. Guidone G. H. M. Schoeps B. L. Silva L. C. . (2024). Genomic insights into a Proteus mirabilis strain inducing avian cellulitis. Braz. J. Microbiol. 55, 4157–4166. doi: 10.1007/s42770-024-01508-6 39235714 PMC11711737

[B9] Elhoshi M. El-Sherbiny E. Elsheredy A. Aboulela A. G. (2023). A correlation study between virulence factors and multidrug resistance among clinical isolates of Proteus mirabilis. Braz. J. Microbiol. 54, 1387–1397. doi: 10.1007/s42770-023-01080-5 37535261 PMC10484824

[B10] Filipiak A. Chrapek M. Literacka E. Wawszczak M. Głuszek S. Majchrzak M. . (2020). Pathogenic factors correlate with antimicrobial resistance among clinical Proteus mirabilis strains. Front. Microbiol. 11, 579389. doi: 10.3389/fmicb.2020.579389 33324365 PMC7723865

[B11] Galani I. Souli M. Panagea T. Poulakou G. Kanellakopoulou K. Giamarellou H. (2012). Prevalence of 16S rRNA methylase genes in Enterobacteriaceae isolates from a Greek university hospital. Clin. Microbiol. Infect. 18, E52–E54. doi: 10.1111/j.1469-0691.2011.03738.x 22264302

[B12] Girlich D. Bonnin R. A. Dortet L. Naas T. (2020). Genetics of acquired antibiotic resistance genes in Proteus spp. Front. Microbiol. 11, 256. doi: 10.3389/fmicb.2020.00256 32153540 PMC7046756

[B13] Guo S. Aung K. T. Tay M. Y. F. Seow K. L. G. Ng L. C. Schlundt J. (2019). Extended-spectrum β-lactamase-producing Proteus mirabilis with multidrug resistance isolated from raw chicken in Singapore: Genotypic and phenotypic analysis. J. Glob. Antimicrob. Resist. 19, 252–254. doi: 10.1016/j.jgar.2019.10.013 31639544

[B14] Harada K. Niina A. Shimizu T. Mukai Y. Kuwajima K. Miyamoto T. . (2014). Phenotypic and molecular characterization of antimicrobial resistance in Proteus mirabilis isolates from dogs. J. Med. Microbiol. 63, 1561–1567. doi: 10.1099/jmm.0.081539-0 25187600

[B15] Hu R. Wang X. Muhamamd I. Wang Y. Dong W. Zhang H. . (2020). Biological characteristics and genetic analysis of a highly pathogenic Proteus mirabilis strain isolated from dogs in China. Front. Vet. Sci. 7, 589. doi: 10.3389/fvets.2020.00589 33134334 PMC7575763

[B16] Humphries R. Bobenchik A. M. Hindler J. A. Schuetz A. N. (2021). Overview of changes to the clinical and laboratory standards institute performance standards for antimicrobial susceptibility testing, M100, 31st edition. J. Clin. Microbiol. 59, e0021321. doi: 10.1128/jcm.00213-21 34550809 PMC8601225

[B17] Hussein E. I. Al-Batayneh K. Masadeh M. M. Dahadhah F. W. Al Zoubi M. S. Aljabali A. A. . (2020). Assessment of pathogenic potential, virulent genes profile, and antibiotic susceptibility of Proteus mirabilis from urinary tract infection. Int. J. Microbiol. 2020, 1231807. doi: 10.1155/2020/1231807 32089693 PMC7029293

[B18] Jain C. Rodriguez-R L. M. Phillippy A. M. Konstantinidis K. T. Aluru S. (2018). High throughput ANI analysis of 90K prokaryotic genomes reveals clear species boundaries. Nat. Commun. 9, 5114. doi: 10.1038/s41467-018-07641-9 30504855 PMC6269478

[B19] Jin Y. Wang Q. Cao R. Sun C. Hu P. Liu Z. . (2024). Preparation and immunological study of a trivalent bacterial ghost vaccine against Escherichia coli, Klebsiella pneumoniae, and Proteus mirabilis in foxes and raccoon dogs. Special Wild Economic Anim. Plant Res. 46, 1–8. doi: 10.16720/j.cnki.tcyj.2024.097

[B20] Li R. He L. Hao L. Wang Q. Zhou Y. Jiang H. (2013). Genotypic and phenotypic characterization of antimicrobial-resistant Escherichia coli from farm-raised diarrheic sika deer in Northeastern China. PloS One 8, e73342. doi: 10.1371/journal.pone.0073342 24039919 PMC3767801

[B21] Liu L. Dong Z. Ai S. Chen S. Dong M. Li Q. . (2023). Virulence-related factors and antimicrobial resistance in Proteus mirabilis isolated from domestic and stray dogs. Front. Microbiol. 14, 1141418. doi: 10.3389/fmicb.2023.1141418 37234544 PMC10206225

[B22] Lv P. Hao G. Cao Y. Cui L. Wang G. Sun S. (2022). Detection of carbapenem resistance of Proteus mirabilis strains isolated from foxes, raccoons and minks in China. Biol. (Basel) 11. doi: 10.3390/biology11020292 35205158 PMC8869598

[B23] Meier-Kolthoff J. P. Auch A. F. Klenk H.-P. Göker M. (2013). Genome sequence-based species delimitation with confidence intervals and improved distance functions. BMC Bioinf. 14, 60. doi: 10.1186/1471-2105-14-60 23432962 PMC3665452

[B24] Meier-Kolthoff J. P. Göker M. (2019). TYGS is an automated high-throughput platform for state-of-the-art genome-based taxonomy. Nat. Commun. 10, 2182. doi: 10.1038/s41467-019-10210-3 31097708 PMC6522516

[B25] Meier-Kolthoff J. P. Sardà Carbasse J. Peinado-Olarte R. L. Göker M. (2022). TYGS and LPSN: a database tandem for fast and reliable genome-based classification and nomenclature of prokaryotes. Nucleic Acids Res. 50, D801–D807. doi: 10.1093/nar/gkab902 34634793 PMC8728197

[B26] Nickel J. C. Wright J. B. Ruseska I. Marrie T. J. Whitfield C. Costerton J. W. (1985). Antibiotic resistance of Pseudomonas aeruginosa colonizing a urinary catheter *in vitro*. Eur. J. Clin. Microbiol. 4, 213–218. doi: 10.1007/BF02013600 3924611

[B27] Pathirana H. De Silva B. C. J. Wimalasena S. Hossain S. Heo G. J. (2018). Comparison of virulence genes in Proteus species isolated from human and pet turtle. Iran. J. Vet. Res. 19, 48–52. doi: 10.22099/ijvr.2018.4768 PMC5960773 29805463

[B28] Ramatla T. Ramaili T. Lekota K. Mileng K. Ndou R. Mphuthi M. . (2024). Antibiotic resistance and virulence profiles of Proteus mirabilis isolated from broiler chickens at abattoir in South Africa. Vet. Med. Sci. 10, e1371. doi: 10.1002/vms3.1371 38357843 PMC10867704

[B29] Sanches M. S. Baptista A. A. S. de Souza M. Menck-Costa M. F. Justino L. Nishio E. K. . (2020). Proteus mirabilis causing cellulitis in broiler chickens. Braz. J. Microbiol. 51, 1353–1362. doi: 10.1007/s42770-020-00240-1 32067208 PMC7455630

[B30] Savage V. J. Chopra I. O'Neill A. J. (2013). Staphylococcus aureus biofilms promote horizontal transfer of antibiotic resistance. Antimicrob. Agents Chemother. 57, 1968–1970. doi: 10.1128/AAC.02008-12 23357771 PMC3623343

[B31] Sun Y. Wen S. Zhao L. Xia Q. Pan Y. Liu H. . (2020). Association among biofilm formation, virulence gene expression, and antibiotic resistance in Proteus mirabilis isolates from diarrhetic animals in Northeast China. BMC Vet. Res. 16, 176. doi: 10.1186/s12917-020-02372-w 32503535 PMC7275385

[B32] Wang Y. Zhang S. Yu J. Zhang H. Yuan Z. Sun Y. . (2010). An outbreak of Proteus mirabilis food poisoning associated with eating stewed pork balls in brown sauce, Beijing. Food Control 21, 302–305. doi: 10.1016/j.foodcont.2009.06.00942574925

[B33] Yin W. Wang Y. Liu L. He J. (2019). Biofilms: The microbial "protective clothing" in extreme environments. Int. J. Mol. Sci. 20, 3423. doi: 10.3390/ijms20143423 31336824 PMC6679078

[B34] Yu Z. Joossens M. Van den Abeele A. M. Kerkhof P. J. Houf K. (2021). Isolation, characterization and antibiotic resistance of Proteus mirabilis from Belgian broiler carcasses at retail and human stool. Food Microbiol. 96, 103724. doi: 10.1016/j.fm.2020.103724 33494897

